# A second generation radiation hybrid map to aid the assembly of the bovine genome sequence

**DOI:** 10.1186/1471-2164-7-283

**Published:** 2006-11-06

**Authors:** Oliver C Jann, Jan Aerts, Michelle Jones, Nicola Hastings, Andy Law, Stephanie McKay, Elisa Marques, Aparna Prasad, Jody Yu, Stephen S Moore, Sandrine Floriot, Marie-Françoise Mahé, André Eggen, Licia Silveri, Riccardo Negrini, Elisabetta Milanesi, Paolo Ajmone-Marsan, Alessio Valentini, Cinzia Marchitelli, Maria C Savarese, Michal Janitz, Ralf Herwig, Steffen Hennig, Chiara Gorni, Erin E Connor, Tad S Sonstegard, Timothy Smith, Cord Drögemüller, John L Williams

**Affiliations:** 1 Division of Genetics & Genomics, Roslin Institute, Roslin, Midlothian, Edinburgh, EH25 9PS, UK; 2 University of Alberta, Edmonton, AB, T6G 2P5, Canada; 3 Laboratoire de Génétique Biochimique et Cytogénétique, INRA-CRJ, 78350 Jouy-en-Josas, France; 4 Istituto di Zootecnica, Università Cattolica del S. Cuore via E. Parmense 84, 29100 Piacenza, Italy; 5 Department of Animal Productions, University of Tuscia, Viterbo, Italy; 6 Department of Vertebrate Genomics, Max Planck Institute for Molecular Genetics, 14195 Berlin, Germany; 7 RZPD German Resource Center for Genome Research, 14059 Berlin, Germany; 8 Parco Tecnologico Padano, via Einstein, Polo Universitario, Lodi 26900, Italy; 9 USDA-ARS, Beltsville Agricultural Research Center, 10300 Baltimore Avenue, Beltsville, MD 20705, USA; 10 USDA-ARS U.S. Meat Animal Research Center P.O. Box 166 Clay Center, NE 68933-0166, USA; 11 Institute for Animal Breeding and Genetics, University of Veterinary Medicine Hannover, Bünteweg 17p, 30559 Hannover, Germany

## Abstract

**Background:**

Several approaches can be used to determine the order of loci on chromosomes and hence develop maps of the genome. However, all mapping approaches are prone to errors either arising from technical deficiencies or lack of statistical support to distinguish between alternative orders of loci. The accuracy of the genome maps could be improved, in principle, if information from different sources was combined to produce integrated maps. The publicly available bovine genomic sequence assembly with 6× coverage (Btau_2.0) is based on whole genome shotgun sequence data and limited mapping data however, it is recognised that this assembly is a draft that contains errors. Correcting the sequence assembly requires extensive additional mapping information to improve the reliability of the ordering of sequence scaffolds on chromosomes. The radiation hybrid (RH) map described here has been contributed to the international sequencing project to aid this process.

**Results:**

An RH map for the 30 bovine chromosomes is presented. The map was built using the Roslin 3000-rad RH panel (BovGen RH map) and contains 3966 markers including 2473 new loci in addition to 262 amplified fragment-length polymorphisms (AFLP) and 1231 markers previously published with the first generation RH map. Sequences of the mapped loci were aligned with published bovine genome maps to identify inconsistencies. In addition to differences in the order of loci, several cases were observed where the chromosomal assignment of loci differed between maps. All the chromosome maps were aligned with the current 6× bovine assembly (Btau_2.0) and 2898 loci were unambiguously located in the bovine sequence. The order of loci on the RH map for BTA 5, 7, 16, 22, 25 and 29 differed substantially from the assembled bovine sequence. From the 2898 loci unambiguously identified in the bovine sequence assembly, 131 mapped to different chromosomes in the BovGen RH map.

**Conclusion:**

Alignment of the BovGen RH map with other published RH and genetic maps showed higher consistency in marker order and chromosome assignment than with the current 6× sequence assembly. This suggests that the bovine sequence assembly could be significantly improved by incorporating additional independent mapping information.

## Background

The global importance of cattle production has resulted in considerable efforts to detect the genes controlling variations in economically important traits. This task is greatly facilitated by the availability of molecular markers ordered along chromosomes. In the last decade a number the bovine genome maps have been published, many of them based on genetic linkage between markers [1-3]. A major disadvantage of linkage maps is that only polymorphic loci can be included, whereas, RH maps can be constructed using sequence information from non-polymorphic loci. Therefore, RH maps potentially contain more coding loci than linkage maps facilitating comparative mapping across species. In contrast to linkage maps, which exploit the frequency of natural recombination between markers to calculate distances and orders of markers, RH maps are constructed using the probability of breaks between markers induced by radiation. Several whole genome radiation hybrid (WGRH) panels are available for cattle that have been used to construct RH maps [4-8]. These RH maps have been used to create comparative maps between bovine and human chromosomes through the alignment of the loci derived from coding sequences [9-15]. The RH maps can also be integrated with other bovine physical maps such as BAC maps constructed by fingerprinting methods by identifying the marker loci within e.g. BAC end sequences [16,17]. This additional mapping information facilitates the ordering of fingerprint contigs and the construction of physical BAC maps covering whole chromosomes. Such physical BAC maps provide a valuable starting point for genome sequencing [18-21]. Fingerprint contig BAC maps have been constructed for cattle using clones from the INRA BAC library [22] and the CHORI-240 BAC library [23]. The ultimate map for a species is the correctly assembled genome sequence. The bovine genome sequencing project started in 2003 and uses a combination of whole genome shotgun sequences (WGS) and sample sequencing of a minimum tiling path of BAC clones spanning the genome. The current, publicly available, bovine genomic sequence (Btau_2.0) has 6-fold genome coverage from WGS assembled into scaffolds and aligned on the chromosomes using limited mapping data. The use of RH and linkage map information [24] would greatly improve the genome sequence assembly [25].

Here we report a second generation RH map of the bovine genome which can be used to improve the construction of an integrated bovine genomic map. Sequences of the markers used to construct the map were aligned with the MARC 2004 linkage map and the Illinois-Texas (ILTX 2005) RH map [15] to investigate discrepancies. Loci that were unambiguously placed in all the maps were then aligned with the Btau_2.0 sequence to identify potential problems in the current sequence assembly.

## Results

### Radiation hybrid map

2735 markers were added to those on the first-generation RH map of Williams *et al*. [7], of which 2473 are newly mapped loci and 262 are previously reported AFLP markers [26], giving a final total of 3966 markers (Table 1). The majority of the new markers, 1999, are within genes, 1072 are microsatellite loci, 262 AFLP markers, 376 BAC end sequences and 257 are from ESTs sequences that do not show convincing similarity to the annotated bovine sequence. The RH chromosome maps constructed from this data can be viewed and information downloaded from the ArkDB database [27].

The total length of the whole genome RH map, including all bovine autosomes and the X chromosome is 760 Rays (R). The map of BTA 28 is the shortest at 1141 cR and the longest is BTA 7 (4408 cR). The average marker interval, over the whole genome, is 19 cR ranging between 12 cR (BTA 29) to 29 cR (BTA 20). Distance comparisons between common markers on the RH map, MARC 2004 linkage map and the bovine sequence suggests, on average, 1 cR on the BovGen RH map is equivalent to 0.04 cM and 23 Kbp, respectively, although this varies considerably across the genome.

### Comparison with the ILTX 2005 RH map

There are 160 marker loci in common between the BovGen RH map described here and the Illinois-Texas (ILTX 2005) RH map [15]. All of these common loci were assigned to the same chromosomes on both maps (see Additional File 1).

**Figure 1:**
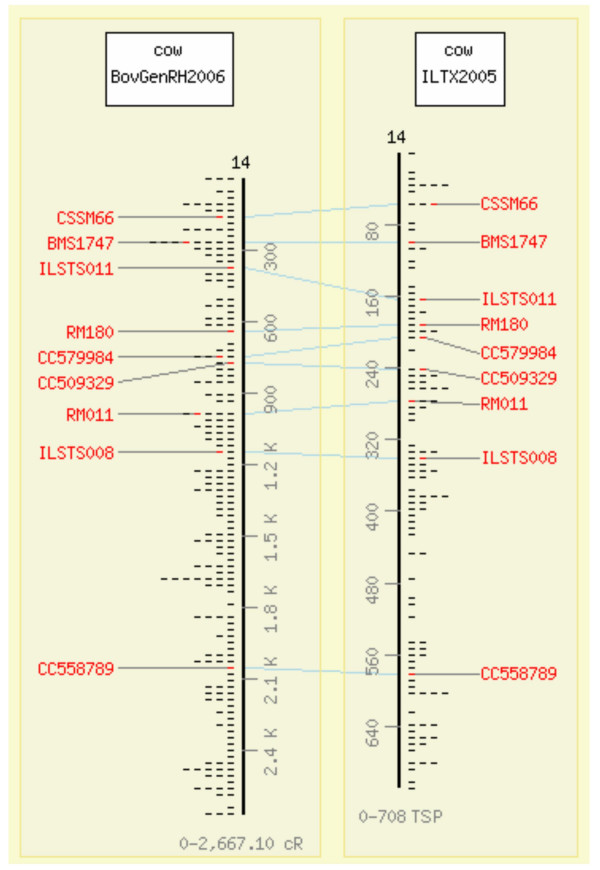
**BovGen RH map of BTA 14 compared with the ILTX 2005 map**. Red dashes represent corresponding marker positions, black dashes non-corresponding positions. Lines between the maps connect markers common in both maps. For an improved legibility only marker names common in both maps are displayed. Distances on the BovGen RH map are scaled in cR, on the ILTX 2005 map in travelling salesman units (TSP).

Three chromosomes (BTA 19, 28 and 29) cannot be assessed for consistency of their order between the ILTX 2005 and BovGen RH map because they have no markers in common. For the remaining 27 chromosomes, 19 are consistent with the BovGen RH map. For example, the BovGen RH map of chromosome 14 has 9 markers in common with the ILTX 2005 map and the order agrees between maps (Figure 1). Out of the 27 chromosomes another 7 have one marker out of 3 to 9 corresponding loci inconsistently positioned. On BTA 8 there are marker order discrepancies between the maps involving 2 out of the 11 corresponding markers.

### Comparison with MARC 2004 linkage map

There are 885 marker loci in common between the BovGen RH and the MARC 2004 linkage map [3] which allows a detailed comparison of map order and chromosome assignment. Inconsistencies in chromosomal assignment are found for 5 of these 885 loci (see Additional File 2). In all these cases only individual markers are involved.

**Figure 2:**
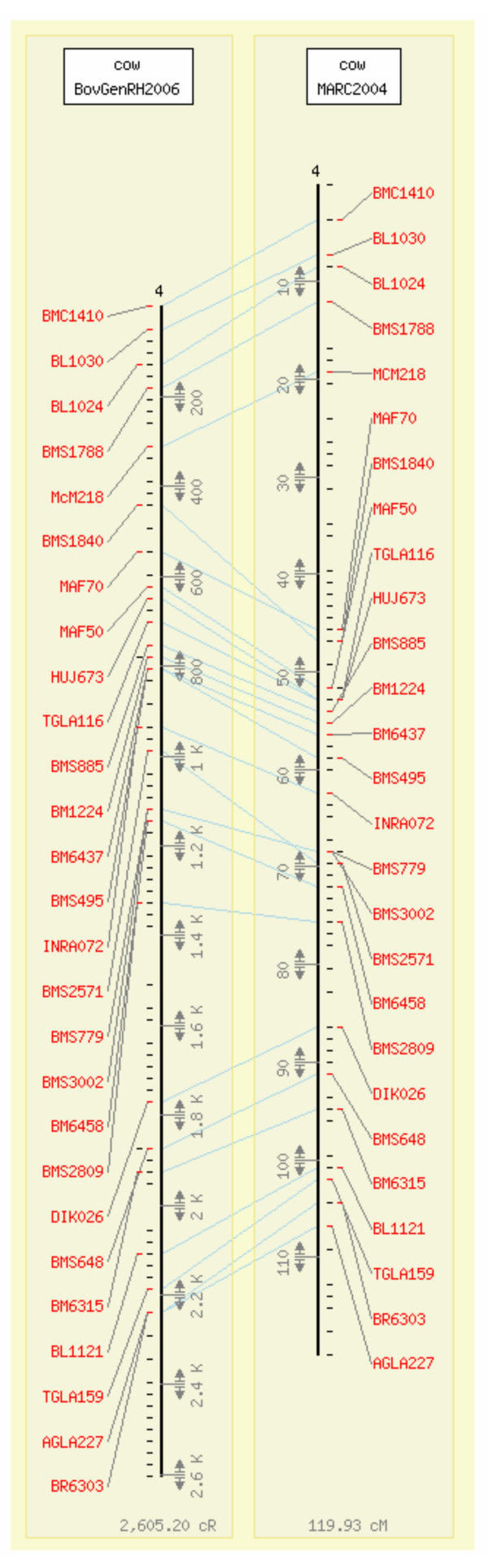
**BovGen RH map of BTA 4 compared with the corresponding MARC 2004 linkage map**. Red dashes represent corresponding marker positions, black dashes non-corresponding positions. Lines between the maps connect markers common in both maps. For an improved legibility only marker names common in both maps are displayed. Distances on the BovGen RH map are scaled in cR, on the MARC 2004 map in centimorgan (cM).

The marker order on 13 chromosomes (BTA 4, 10, 11, 13, 14, 16, 18, 21, 23, 24, 25, 27 and 28) is in very close agreement between the BovGen RH and MARC 2004 maps. For example, the order of the 27 markers on BTA 4 that are in common show only minor inversions of two pairs of linked loci: *BMS1840 *and *MAF70 *appear in different order and *BMS2571 *is located telomeric to *BMS779 *and *BMS3002 *on the BovGen RH map, but centromeric on the MARC 2004 genetic map (Figure 2). Despite the similarity the marker order as suggested by the MARC 2004 map is inconsistent with the multipoint map BovGen RH data. If the RH data is forced into the order of the MARC 2004 map a much lower probability for the map is obtained. Thus, in order to determine the true order of these markers additional information is required.

**Figure 3:**
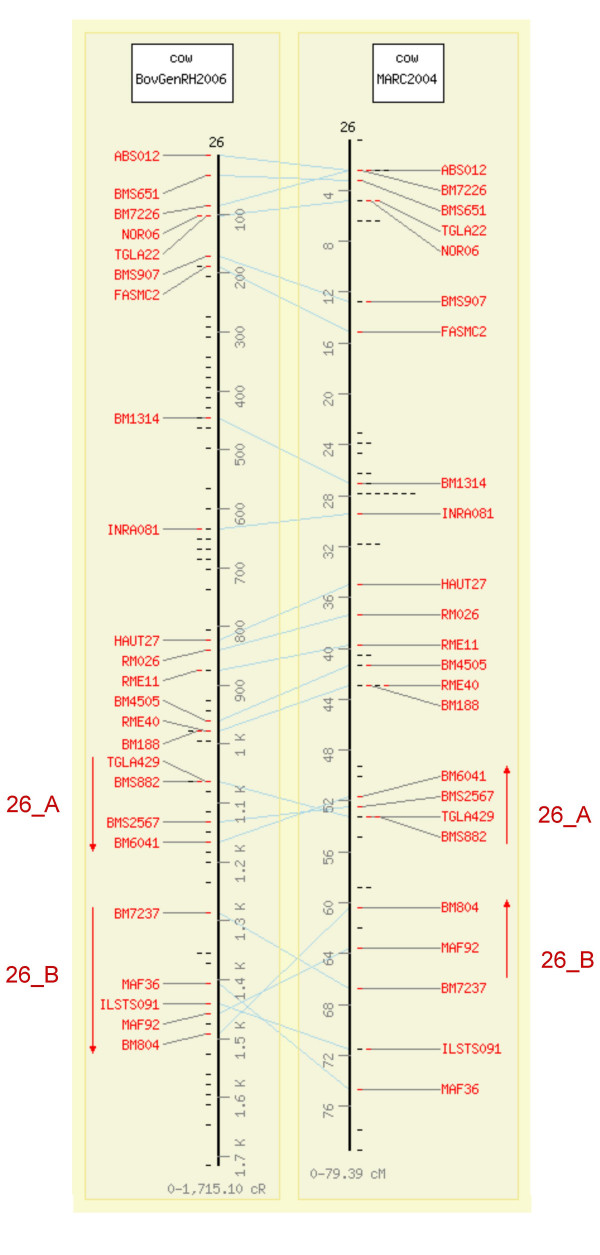
**BovGen RH map of BTA 26 compared with the corresponding MARC 2004 linkage map**. Red dashes represent corresponding marker positions, black dashes non-corresponding positions. Lines between the maps connect markers common in both maps. For an improved legibility only marker names common in both maps are displayed. Locations of discussed marker groups and their orientation are indicated by arrows. Distances on the BovGen RH map are scaled in cR, on the MARC 2004 map in cM.

On a further 13 chromosomes, minor discrepancies between these maps were observed. On BTA 3, 5, 8, 9, 12, 17, 19, 22 and X the order of markers is essentially the same, but with a number of individual markers at different positions. For BTA 1, 2, 6 and 26 differences were observed involving the orientation of groups of markers, but with a conserved order of markers within the group. For example, on BTA 26 the marker order is in general consistent between the BovGen RH and the MARC 2004 linkage map, however two small groups of linked markers 26_A (*BMS882*, *TGLA429*, *BMS2567 *and *BM6041*) and 26_B (*MAF36*, *ILSTS091*, *MAF92 *and *BM804*) have the same marker order in both maps, but are inverted with only one marker (*BM7237*) at a divergent position (Figure 3).

**Figure 4:**
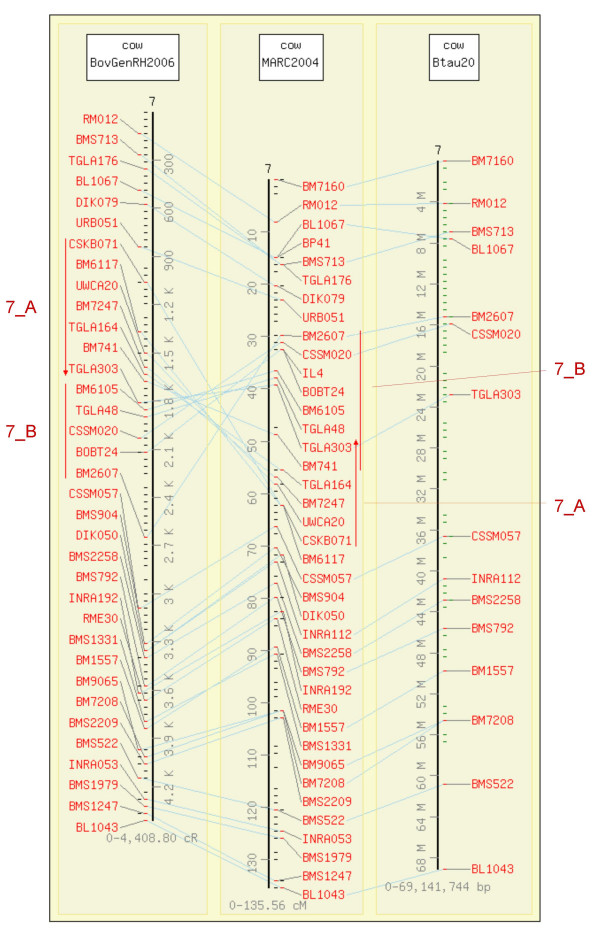
**BovGen RH map of BTA 7 (left) compared with the corresponding MARC 2004 linkage map and Btau_2.0 (right)**. Red dashes represent corresponding marker positions, black dashes non-corresponding positions. Lines between the maps connect markers common in both maps. For an improved legibility only marker names common in both maps are displayed. Locations of discussed marker groups are marked and the orientation of the group 7_A indicated by arrows. Distances on the BovGen RH map are scaled in cR, on the MARC 2004 map in cM.

On four chromosomes major inconsistencies are observed where groups of linked markers map to different chromosomal positions (BTA 7, 29) or where the order of markers differs within several marker groups (e.g. BTA 7, 15 and 20). On BTA 7, for example, the position of two groups of linked markers 7_A (limited by the markers *CSKB071 *and *TGLA303*) and 7_B (limited by the markers *BM6105 *and *BM2607*) are exchanged. In addition the group 7_A is in a different orientation in both maps, while the marker order in 7_B is inconsistent (see Figure 4). Nevertheless, these discrepancies only involve about a quarter of the chromosome, and 12 out of the 38 common markers. The map positions of the other 26 markers are in close agreement between the two maps. These differences could be further investigated by comparison with additional mapping information (see below).

### Comparison with the 6× bovine assembly

Of the 3966 markers successfully included in the RH map, 2898 could be unequivocally assigned to a position in the Btau_2.0 bovine sequence, 2767 were assigned to the same chromosome, but 131 mapped on different chromosomes between the BovGen RH map and the sequence (Additional File 3). On seven chromosomes inconsistent assignments involving groups of three or more markers were observed (Table 2).

On all but two chromosomes (BTA 9 and 14) there were many differences between the map order and the sequence: on many chromosomes large discrepancies involving groups of linked markers and/or large numbers of individual loci were seen, particularly on chromosomes 5, 7, 16, 22, 25 and 29. For example, on chromosome 7 two large groups of linked loci on the BovGen RH map locate to divergent positions in both the Btau_2.0 and MARC 2004 map, however, these latter two maps agree. This inconsistency is similar to an inconsistency observed between the BovGen RH and the MARC 2004 map, resulting in a good agreement with the Btau_2.0 sequence (Figure 4). Further information from the ILTX 2005 map does not help to resolve this inconsistency as there is only one marker in common with the BovGen RH map in this region.

**Figure 5:**
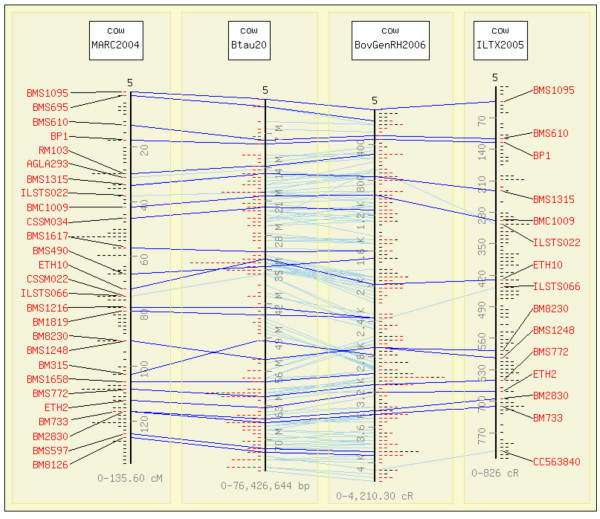
**BovGen RH map of BTA 5 compared with MARC 2004 (far left), Btau_2.0 (left), and the ILTX 2005 map (right)**. Only labels of corresponding markers of the MARC 2004 and ILTX 2005 map are displayed. Lines between the maps connect common markers. Correspondences of consistently ordered markers included in Btau_2.0 and BovGen RH are highlighted by dark blue. Red dashes represent corresponding marker positions, black dashes non-corresponding positions. Distances on the Btau_2.0 are scaled in Mbp. Distances on Btau_2.0 are scaled in Mbp, on the BovGen RH map in cR, on the MARC 2004 map in cM and on the ILTX 2005 map in TSP.

When markers that were at inconsistent positions between the BovGen RH and either the ILTX 2005 or MARC 2004 linkage map were removed, 150 common markers with the ILTX 2005 map and 771 common markers with the MARC 2004 linkage map remained. The mapping order of these markers was then compared with the order in the bovine sequence. The comparison with the Btau_2.0 sequence still revealed discrepancies across the whole genome. For example, on BTA 5 four markers which could be assigned to positions in the sequence assembly appeared to have inconsistent positions (*AGLA293*, *ILSTS022*, *CSSM022*, *ILSTS066*) when compared between the BovGen RH, the MARC 2004 and/or the ILTX 2005 map. After their removal the remaining corresponding markers are in close agreement between the three maps but still reveal inconsistencies with the sequence assembly (Figure 5). Many of the markers which are in common between the BovGen RH map and Btau_2.0 are not present in the MARC 2004 map. These markers tend to have a higher inconsistency when compared to the sequence assembly.

## Discussion

The ability to determine the order of close markers on genome maps differs between approaches, and all approaches, including the assembly of a whole genome sequence, are prone to errors. In some cases insufficient information is available to assign the correct order or positioning of loci, while data errors can introduce distortions in the maps. The ultimate genome map of a species is the correctly ordered DNA sequence. Achieving the correct sequence assembly uses several sources of information. Sequence information from other species, including the human genome could be used as a template, but this approach should be treated with extreme caution as species specific variations are known [28]. Therefore, direct sequence information is used for the local assembly of shotgun sequence reads into contigs, and these contigs are then assembled into scaffolds using additional information, such as overlapping clones, and sequences from paired clone ends. The ordering of these scaffolds on chromosomes and assembly of the final sequence relies on additional mapping information, including BAC fingerprint contig maps, linkage maps and RH maps. In this paper we describe an RH map with almost 4000 mapped loci which will contribute to the assembly of the bovine genome sequence.

### Comparison with other linkage and RH maps

The consistency in ordering of common loci can be assessed across different maps, however, it is important that the information used when assembling the maps is independent, as circular arguments can give a false measure of agreement. The approach of e.g. Itoh *et al. *[8] was to use the linkage map as template for their RH map; in contrast we did not use any prior information to construct the RH map presented here. This was because the aim was to assemble the most likely map using completely independent data and so not to propagate potential errors across different maps. Resolving these inconsistencies often requires the use of additional independent evidence such as BAC FPC mapping data or cytogenetic (fluorescent in-situ hybridisation, FISH) information. We carried out an alignment of the BovGen RH map with the other available bovine genome maps and the Btau_2.0 sequence assembly, but only after the RH maps had been constructed. While this approach relies on only one source of information it may not result in the "best" possible map, however, it avoids bias and the resulting independent map can then be used to develop a combined map which carries a measure of map confidence based on similarity and differences between maps.

The BovGen and ILTX 2005 RH map appear to be more consistent with each other than with the MARC 2004 linkage map. Some inconsistencies between linkage and RH maps may be due to the different mapping approaches. However, the observation of the apparently higher consistency between the RH maps must be treated with care as the BovGen RH map has fewer loci in common with the ILTX 2005 map than with the MARC 2004 linkage map, and so fewer discrepancies could be detected. The ILTX 2005 map was constructed on the basis of the first-generation map (ILTX 2004) by increasing the marker density and a subsequent rigorous removal of markers which did not pass a quality control procedure [15]. In this process a significant number of markers common to both the BovGen RH and the ILTX 2004 map were removed and as a result there are fewer correspondences of our map with ILTX 2005 than with the old ILTX 2004. It can be assumed that this process improved the ILTX map enormously. Therefore, we used the ILTX 2005 map for comparison despite fewer correspondences. Nevertheless, the ILTX 2004 map has a similarly high marker order consistency and chromosomal assignment with the BovGen RH map (data not shown).

### Comparison with the sequence assembly

Sequence similarity search algorithms used to align maps with Btau_2.0 have a considerable risk of errors as they may also detect gene duplications or similar motifs in different genes. To minimize this problem we used very stringent parameters for minimum homology and maximized the required length of overlap between sequences. In addition, sequence matches were assessed manually. Thus the loci we aligned between the different maps and the bovine sequence carry a very high probability of correctly assigned homology. Differences in the position of individual markers between different maps could be the result of technical variations explained by using different parameters and algorithms to construct the multipoint maps. Inconsistencies in the chromosomal assignment of individual markers may also have simple explanations, such as poor primer design resulting in amplification of related loci and not the target locus. Of greater importance for the interpretation of the map information are inconsistencies affecting whole groups of linked markers. To minimise the propagation of errors in individual maps we eliminated individual markers that were inconsistently placed between BovGen RH, ILTX 2005 and MARC 2004 maps from a further combined analysis against the sequence assembly.

While the BovGen RH map is in general agreement with the ILTX map and the MARC 2004 map, there is poor agreement with the Btau_2.0 sequence at specific chromosomal regions. In such regions, e.g. those described above on chromosomes 7, 25 and 29, the assembled Btau_2.0 sequence is most consistent with the linkage map. This is not surprising, because, among other sources of information, the MARC 2004 map was used to order the sequence scaffolds in Btau_2.0. Recalculating the BovGen data for these chromosomes and forcing the markers into the order they appear in the sequence assembly significantly increases the map length and reduces the probability showing that our data are not consistent with the sequence order. Further information must be generated to resolve such inconsistencies.

### Assignment of markers to different chromosomes

A problem in the genome assembly is that of erroneous assignments of sequence scaffolds. By comparing assignments among the different RH and linkage maps [1,39,40] and also using comparative human [30,31,41-45] or mouse [29] information, it seems likely that the assignment in the bovine assembly is most often at fault (Table 2). For example the markers *PTK2B*, *BZ948637 *and *B4GALT1 *(Table 2, case 4) are closely linked on the BovGen RH map of BTA 8 and the linkage map of Barendse *et al. *[1] which also places these genes on BTA 8. This is also consistent with data from Fiedorek & Kay [29] who mapped *PYK2B *(alias *PTK2B *or *Fadk*) on murine chromosome 15 and Inazawa *et al. *[30] who mapped the gene on human chromosome 8 at positions which share conservation of synteny with BTA 8 [15]. However, these marker loci are placed on chromosome 5 in the Btau_2.0 sequence assembly. All three markers are located on a single sequence scaffold (chr5.80), suggesting that the chromosomal assignment of this scaffold is likely to be incorrect.

A group of neighbouring markers formed by *KIAA0284*, *Q9Y4F5*, *KNS2 *and *BTBD6 *were assigned to BTA 11 on the BovGen RH map; however, this assignment is not consistent with other mapping data (Table 2, case 5). The human homologues of these loci are located on human chromosome 14 [31], suggesting that this group is correctly assigned in the Btau_2.0 sequence to chromosome 21 and that in this case the BovGen RH assignment is incorrect. Nevertheless, the linkage of this group to other markers on BTA 11 is convincing with LOD linkage values up to 13.8 between the extreme marker *KIAA0284 *and the neighbouring markers on the BovGen RH map. If this marker group is tested with the markers located on BTA 21 using the BovGen RH datasets it shows no linkage. In the Btau_2.0 assembly this marker group is at an extreme telomeric position which suggests that the statistical support for this assignment is weak. This chromosomal assignment may have been made on the expected position derived from the supposed conservation of synteny between human and cattle chromosomes and should be tested using independent evidence.

The markers *BZ850749*, *CC517527 *and *CC471629 *are assigned to BTA 14 on the BovGen RH map and to BTA 25 in the Btau_2.0 sequence assembly (Table 2, case 6). These markers are derived from BAC end sequences of clones from the CHORI-240 library and are not present on other maps. All these markers are assigned to the scaffold Chr25.84 and are in a chromosomal region of the assembly with a low density of corresponding markers. In contrast on the BovGen RH map, the markers in the same region are at a higher density. This suggests that these markers are more tightly linked on the BovGen RH map. No further information is available to resolve this inconsistency.

Independent information is essential to produce the best maps of the bovine genome and to assemble the most accurate sequence. In addition to the RH mapping approach and linkage mapping that have been discussed here the refinement of the sequence should use additional sources of information such as BAC FPC maps, comparative mapping, fluorescent in situ hybridization, and somatic cell hybrid mapping.

## Conclusion

There is reasonable consistency between the RH map presented here, the MARC 2004 linkage map and the ILTX 2005 map. However, where the maps differed it is usually not possible to determine which order of markers is correct. Manipulating the data to make the different maps match is not productive. When the major discrepancies are removed a number of inconsistencies with the Btau_2.0 bovine sequence assembly still remain. Using the various mapping information it is possible to identify potential errors in the assembly of the current bovine genome sequence which should be investigated further to aid the improvement of the next sequence build.

Using the information presented here it will not be possible to reach a final version of the sequence. The Btau_2.0 sequence assembly contains more than 100,000 scaffolds of which only 4409 are anchored to chromosomes using markers from the genetic map, and about half of the anchored scaffolds contain two mapped markers allowing them to be orientated. The data presented here will increase the number of scaffolds that can be assigned and orientated. Nevertheless it will be necessary to use additional information such as fingerprinting or BAC skim data and physical maps, such as FISH based techniques, which in addition to comparative mapping data will help to finalize the assembly and yield a reliable sequence.

## Methods

### Sequencing of ESTs

A non-redundant "unigene" set of ESTs was selected by oligo-nucleotide fingerprinting and clustering of cDNAs from a brain library (Herwig *et al.*, manuscript in preparation). This non-redundant cDNA clone set contains 23040 bovine clones grouped by sequence assembly of ESTs into 14989 unique cDNA clusters and singletons. The cDNA clones of the "unigene" set were amplified in a 384-well microplate format by PCR consisting of an initial denaturing for 2 min at 95°C, denaturing for 45 sec at 94°C, annealing and elongation for 4 min at 65°C in 30 cycles. PCR primers were complementary to the insert-flanking vector sequences. The PCR mix contained 5 pmol forward primer (GGA TCT ATC AAC AGG AGT CCA AGC TCA GCT), 5 pmol reverse primer (TCA CCA TCA CGG ATC CTA TTT AGG TGA CAC), 0.1 mM dNTP's, 1.5 M Betain, 1× PCR buffer, 0.1 mM Cresol Red and 1 U per reaction Taq DNA polymerase. PCR buffer consisted of 0.5 M KCl, 1% Tween20, 15 mM MgCl_2_, 350 mM TrisBase, 150 mM Tris/HCl pH 8.3. PCR fragments were subjected to sequence analysis using BigDye-terminator chemistry (Applied Biosystems) and a 3700 DNA sequencer (Applied Biosystems). Average sequence read length was 750 bp. The individual EST sequence data were submitted to GenBank and are publicly available under accession numbers CO871676–CO897060.

### Primer design

Maximum sequence information for annotation was achieved by aligning the EST data with available public cattle transcript sequences contained in the TIGR bovine gene index. TIGR clusters and corresponding ESTs cattle sequences produced here were aligned and the resulting 14989 cluster sequences (consensus) used for the subsequent construction of primers. Cluster sequences were aligned with bovine genomic sequences and only those showing clear splicing were used to define the precise exon-intron boundaries for the final primer selection (see below).

The primer design was carried out using dedicated software now in the public domain [32]. The software uses the nearest-neighbour method [33] to predict the complimentarily of primers and secondary structures (dimers, hairpin etc.) and is able to process large number of sequences in batches, picking primers in designated regions. To minimize the amplification of hamster DNA contained within the RH panel cell lines, primer pairs were designed with one primer within exon, the other within the adjacent intron or non-coding sequence.

The primer design was standardized to achieve a maximum of uniformity in amplification conditions. Primer details are available to the public in the ArkDB database [27].

### Screening of the Roslin RH panel

2473 marker loci were successfully typed on the 94 cell lines of a 3000-rad bovine/hamster RH panel as described by Williams *et al. *[7]. Vectors of 262 AFLP markers [26] were added to the dataset. Resulting vectors for the 3966 marker loci used (including 1231 previously mapped loci [7]) are available in the Additional File 4 for download.

### RH data analysis

RH vectors were assigned to chromosomes by analysing 2-pt linkage with mapped loci [7] using RH mapper [34]. Multipoint maps were constructed using the default algorithm of the Carthagene software [35]. The initial multi-point map was improved by an iterative process of inspection of marker loci and removal and alternative addition of badly linked or disrupting loci. This process resulted in the removal of 122 loci that could not be reliably fitted into the chromosome maps with the highest probability. The best maps generated by this process were compared to the ComRad RH-map [7] and the MARC 2004 linkage map [3] and regions showing discrepancies examined in detail to identify the presence of problem markers. Marker positions on the maps are available from the ArkDB database [27].

### Mapping of marker associated sequences against the bovine sequence assembly

ESTs sequences used to design the primers for mapped loci were aligned with the assembled 6× bovine sequence assembly (Btau_2.0) using BLAST [36] and SPIDEY [37]. To filter out incorrect alignments the BLAST e-value was set to a maximum of 1e-20 and minimum percent identity to 90%. In addition, the relative length of the BLAST hit (i.e. coverage, or length of the hit divided by the length of the query sequence) had to be at least 80%. Where ambiguous alignments were observed higher stringency filters were applied (sequence similarity higher than 97.5% and coverage higher than 90%).

### Diagrammatic representation of chromosomal maps

Visual representation of map alignments was achieved using cMap [38].

## Authors' contributions

OJ screened the RH panel, analysed RH data, constructed chromosome maps, performed map and sequence comparisons and drafted the manuscript. JA aligned sequences against the bovine assembly, designed the BovGen RH database and adapted the visualisation of the results. NH performed data analysis. SMK screened the RH panel, performed data analysis and constructed chromosome maps. MJ, EM, AP, JY, SF, LS, MFM, CM and MCS performed screening of the RH panel. AL created associated web based resources. AE performed data analysis and integrated RH and BAC data. PAM, RN, CG and EM produced AFLP and EST data on the RH panel and constructed chromosome maps. AV performed primer design. MJA, RH and SH sequenced the set of ESTs, managed EST sequences and performed sequence analysis. CG screened the RH panel for AFLP markers. CD, EEC and TSS developed EST markers and screened the RH panel. SM provided the loci mapped and oversaw the genotyping and data analysis at the University of Alberta. TSM provided primers and linked EST sequences to locus names. JLW provided the RH panel, initiated the project and was responsible for the overall design of the study, oversaw the work, performed 2-pt and some multipoint analysis, managed RH data and contributed to drafting and editing the manuscript.

All authors read and approved the final manuscript.

## Supplementary Material

Additional File 1 **Matrix of common markers of the BovGen RH map with the ILTX 2005 map**. Chromosomes on the BovGen RH map (vertical) and the ILTX 2005 map (horizontal) are indicated in bold. Numbers indicate the number of common markers between the BovGen RH map and the ILTX 2005 map. Numbers in the diagonals are the number of markers assigned to the same chromosomes. Numbers out of the diagonal are markers assigned to different chromosomes in the BovGen RH map and the ILTX 2005 map. Click here for file

Additional File 2 **Matrix of common markers of the BovGen RH map with the MARC 2004 linkage map**. Chromosomes on the BovGen RH map (vertical) and the MARC 2004 linkage map (horizontal) are indicated in bold. Numbers indicate the number of common markers between the BovGen RH map and the MARC 2004 linkage map. Numbers in the diagonals are the number of markers assigned to the same chromosomes. Numbers out of the diagonal are markers assigned to different chromosomes in the BovGen RH map and MARC 2004 linkage map. Click here for file

Additional File 3 **Matrix of common markers of the BovGen RH map with the Btau_2.0 sequence**. Chromosomes on the BovGen RH map (vertical) and Btau_2.0 sequence assembly (horizontal) are indicated in bold. Numbers indicate the number of common markers between the BovGen RH map and the Btau_2.0 sequence assembly. Numbers in the diagonals are the number of markers assigned to the same chromosomes. Numbers out of the diagonal are markers assigned to different chromosomes in the BovGen RH map and the Btau_2.0 sequence assembly. Click here for file

Additional File 4 **BovGen RH vectors**. The first column indicates the marker name, the second column indicates the assigned chromosome and the third column gives RH vectors used for map construction. The vectors give screening results for each the used 94 cell lines for the Roslin RH panel. "0" indicates that the fragment is not present in the cell line, "1" indicates presence of the fragment in the cell line, and "2" indicates ambiguous results. Click here for file

## Figures and Tables

**Table 1:** Statistics of the BovGen RH map by chromosome

**BTA**	**marker numbers**											
	
	**no of markers**	**new mapped**	**AFLP [26]**	**BES**^a^	**genes**	**micro-satellites**	**unknown ESTs**^b^	**cR**	**cM**	**Mbp**	**cR/marker**	**Mbp/marker**
**1**	157	90	8	2	69	70	8	3695.8	154.67	102.83	23.39	0.65
**2**	169	123	11	3	98	46	11	3487.7	128.88	86.54	20.64	0.51
**3**	208	172	10	3	134	35	26	4405.4	128.9	85.36	21.39	0.41
**4**	110	63	10		64	36		2605.2	119.93	69.56	23.05	0.62
**5**	276	230	9	94	105	40	28	4210.3	135.6	76.43	15.31	0.28
**6**	59	34	6		33	20		1699.4	134.42	69.62	28.8	1.18
**7**	217	166	13		141	45	18	4408.8	135.56	69.14	20.32	0.32
**8**	94	59	7	10	35	38	4	2458.6	128.62	62.12	26.44	0.67
**9**	133	70	8		58	64	3	3004.1	116.17	64.65	22.59	0.49
**10**	160	123	17	2	91	40	10	2760.5	118.83	70	17.04	0.43
**11**	177	136	14	3	102	48	10	3658.9	130.97	87.17	20.56	0.49
**12**	89	53	16	1	34	36	2	1553.3	109.95	48.61	17.45	0.55
**13**	128	90	14		64	36	14	2275.4	105.38	62.72	17.78	0.49
**14**	222	189	1	169	16	33	3	2667.1	103.95	50.71	11.96	0.23
**15**	147	55	12	2	77	38	18	2434.1	109.75	53.82	16.56	0.37
**16**	121	89	9	1	72	33	6	2957.2	94.46	56.99	24.64	0.47
**17**	115	80	15		68	27	5	2654.1	95.86	45.92	23.08	0.4
**18**	200	164	9	5	141	25	20	3182.1	84.38	56.51	15.91	0.28
**19**	156	111	7	53	58	35	3	2136.7	109.61	56.39	13.7	0.36
**20**	58	35	9		26	23		1712.2	82.94	42.95	29.52	0.74
**21**	62	27	9		18	33	2	1449	83.79	49.72	23.37	0.8
**22**	111	79	5	5	69	31	1	2031.9	88.1	48.33	18.31	0.44
**23**	129	85	8	1	80	20	20	2345.7	80.05	41.6	18.04	0.32
**24**	68	21	6	5	19	35	3	1588.7	78.13	45.25	23.36	0.67
**25**	136	102	6	1	89	31	9	2256.2	68.42	41.41	16.96	0.31
**26**	75	40	6		36	30	3	1715.1	79.39	35.72	22.87	0.48
**27**	65	34	3	11	18	29	4	1191.2	67.37	31.57	18.33	0.49
**28**	65	36	5	4	25	22	9	1141.3	61.66	34.89	17.56	0.54
**29**	149	113	4		103	34	8	1895.7	69.73	45.82	12.64	0.31
**X**	110	66	5	1	56	39	9	2390.7	146.5	47.9	22.14	0.44
	
**Total**	**3966**	**2473**	**262**	**376**	**1999**	**1072**	**257**	**75972.4**	**3151.97**	**1740.25**	**19.16**^c^	**0.44**^c^

^a ^BAC end sequences

^b ^ESTs which could not be assigned to an annotated sequence

^c ^average over whole genome

**Table 2:** Inconsistent chromosome assignments between the BovGen RH map and Btau_2.0 sequence. Only the seven most significant cases are listed, involving at least three linked markers.

Case	Markers involved	Assignment Bodge RH BTA	Assignment Btau_2.0 BTA	Other assignments species, chromosome [reference]
1	*BMS4030* *BOVGEN_158* *BOVGEN_91*	**1**	4	**BTA 1 **[39]
2	*MAF23* *BZ855103* *BZ864360*	**5**	4	**BTA 5 **[40]
3	*ADH7* *ADH1A* *ADN1C*	**6**	21	**HSA 4**^a ^[42, 42, 43]
4	*PTK2B* *BZ948637* *B4GALT1*	**8**	5	**BTA 8 **[1], **MMU 15**^a ^[29], **HSA 8**^a ^[30]
5	*KIAA0284* *Q9Y4F5* *KNS2* *BTBD6*	11	**21**	**HSA 14**^a ^[31]
6	*BZ850749* *CC517527* *CC471629*	14	25	-
7	*ACLY* *KLHL11* *SC65* *JUP* *E0362G17*	**19**	23	**HSA 17**^a ^[44, 45]

^a ^HSA 4 is a homologue to BTA 6, MMU 15 and HSA 8 to BTA 8, HSA 14 to BTA 21 and HSA 17 to BTA 19 [15]. Most likely assignments are indicated by bold font.
